# Virulence-related O islands in enterohemorrhagic Escherichia coli O157:H7

**DOI:** 10.1080/19490976.2021.1992237

**Published:** 2021-10-28

**Authors:** Lingyan Jiang, Wen Yang, Xinlei Jiang, Ting Yao, Lu Wang, Bin Yang

**Affiliations:** a TEDA Institute of Biological Sciences and Biotechnology, Nankai University, TEDA, Tianjin, P. R. China; b School of Environmental and Municipal Engineering, Tianjin Chengjian University, Tianjin, P. R. China

**Keywords:** Enterohemorrhagic *Escherichia coli* O157:H7, O island, genomic island, virulence, adherence

## Abstract

Enterohemorrhagic *Escherichia coli* (EHEC) O157:H7 is a principally foodborne pathogen linked to serious diseases, including bloody diarrhea, hemorrhagic colitis, and hemolytic uremic syndrome. Comparative genomics analysis revealed that EHEC O157 contains 177 unique genomic islands, termed O islands, compared with the nonpathogenic *E. coli* K-12 laboratory strain. These O islands contribute largely to the pathogenicity of EHEC O157:H7 by providing numerous virulence factors, effectors, virulence regulatory proteins, and virulence regulatory sRNAs. The present review aimed to provide a comprehensive understanding of the research progress on the function of O islands, especially focusing on virulence-related O islands.

## Introduction

Enterohemorrhagic *Escherichia coli* (EHEC) O157:H7 is an important human pathogen that specifically colonizes the large intestine, causing disease.^1^^, 2^ Shiga toxins (Stxs), which are the major virulence factors of EHEC O157:H7, are known to cause damage to a variety of cell types and have often been associated with hemorrhagic colitis (HC) and the lethal hemolytic uremic syndrome (HUS) in humans.^3,4^ The pathogenesis of EHEC O157:H7 infections is characterized by the formation of an attaching and effacing (A/E) lesion that involves the intimate attachment of bacteria to the host enterocyte membrane, the subversion of actin and cytoskeletal components, and the formation of a pedestal structure beneath the adherent bacteria.^5,6^ The ability of EHEC O157:H7 to form A/E lesions is conferred by a large pathogenicity island, termed locus of enterocyte effacement (LEE), which consists of five polycistronic operons (LEE1 to LEE5).^7^ In particular, LEE encodes a type III secretion system (T3SS) that exports effector molecules, including the intimin adhesin, the translocated intimin receptor (Tir), and several secreted proteins (Esp), which are important in the modification of the host cell signal transduction during the formation of A/E lesions.^1,8^

**Figure 1.**
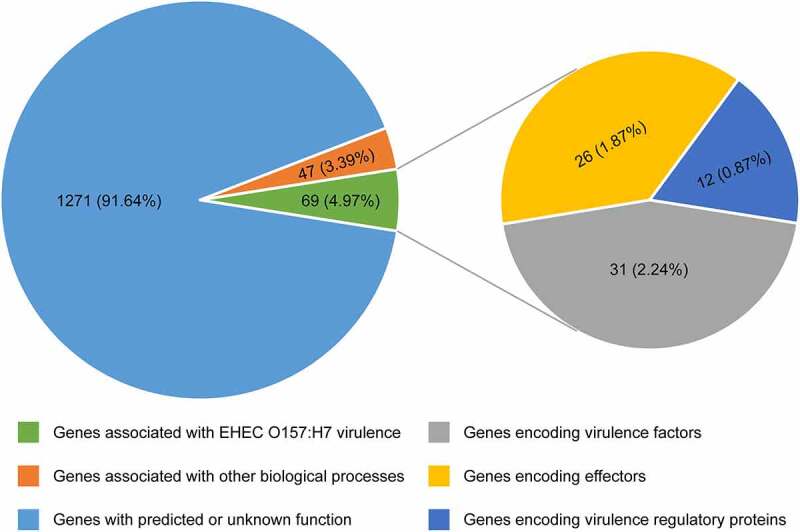
Pie chart showing the functional classification of 1387 OI genes. Each pie slice represents a major functional group of genes. Values represent the number (and percentage) of genes in a particular category

Comparative genomic analyses revealed that the EHEC O157:H7 strain EDL933 contains 177 genomic islands, termed O islands (OIs), which are absent from the genome of the nonpathogenic *E. coli* K-12 MG1655. These OIs encompass 1387 genes (26% of the total), with most of them encoding hypothetical albeit as-yet-uncharacterized proteins.^9,10^ Studies on the function of OIs have made substantial progress during the last 2 decades, and a growing number of OI-associated genes have been assigned a function. Among these 1387 OI genes, 69 genes (4.97%) are associated with EHEC O157:H7 virulence, including 31 genes (2.24%) encoding virulence factors, 26 genes (1.87%) encoding effectors, and 12 genes (0.87%) encoding virulence regulatory proteins; 47 genes (3.39%) are associated with other biological processes; the function of remaining 1271 genes (91.64%) has been predicted or is unknown (Figure 1). In the present review, we aimed to provide a broad overview of the function of OIs, especially focusing on OIs associated with the virulence and pathogenicity of EHEC O157:H7. For convenience, we grouped these OIs into four main categories according to the function of genes contained in these OIs: (1) OIs encoding virulence factors, (2) OIs encoding effectors, (3) OIs encoding virulence regulatory proteins, and (4) OIs encoding virulence regulatory sRNAs.

## OIs encoding virulence factors

**Figure 2.**
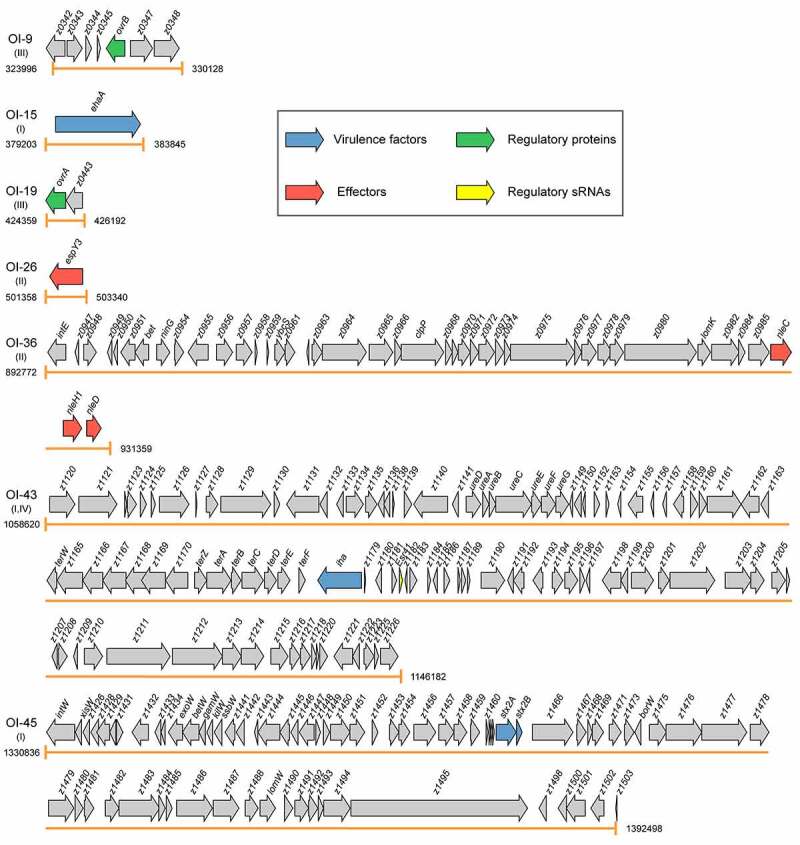
Graphical representation of the gene content and organization of virulence-related OIs. Arabic numerals represent the starting and ending positions of OIs in the EHEC O157:H7 strain EDL933 genome (GenBank accession no. AE005174). Roman numerals represent the four categories of virulence-related OIs: I = OIs encoding virulence factors, II = OIs encoding effectors, III = OIs encoding virulence regulatory proteins, and IV = OIs encoding virulence regulatory sRNAs. Arrows represent genes, which are color-coded to indicate functions: blue = genes encoding virulence factors, red = genes encoding effectors, green = genes encoding virulence regulatory proteins, yellow = genes encoding virulence regulatory sRNAs, and gray = genes not related to EHEC O157:H7 virulence or are with unknown functions

Virulence factors, encoded by pathogenicity islands, plasmids, and prophages, are necessary for colonization and replication within the host, neutralization of host defenses, and spread into new hosts.^11^ The main virulence determinants of EHEC O157:H7 include adhesins, T3SS that inject effector proteins into host cells, Stxs, and iron acquisition systems.^12^ To date, eight OIs, namely OI-15, OI-43, OI-45, OI-48, OI-57, OI-93, OI-122, and OI-148, have been reported to encode virulence factors (Figure 2 and Table 1).

**Table 1.** Virulence-related O islands in EHEC O157:H7

O islands	Proteins	Functional types	Virulence-related functions	References
O islands containing virulence factors				
OI-15	AID15	Virulence factor	AID15, as an adhensin, promotes biofilm formation and adhesion to host epithelial cells.	^13,14^
OI-43	Iha	Virulence factor	Iha, as an IrgA adhesion homolog, promotes EHEC O157:H7 adhesion to host epithelial cells and increases its colonization in the ligated pig intestine.	^15^
OI-45	Stx2A Stx2B	Virulence factor	Stx2A and Stx2B are the 2 subunits of Shiga toxin 2. Shiga toxin inhibits host protein synthesis and increases EHEC O157:H7 survival in macrophages.	^16–18^
OI-57	AdfO	Virulence factor	AdfO resembles Paa, a virulence-associated protein of porcine enteropathogenic *E. coli*. AdfO promotes EHEC O157:H7 adherence to HeLa cells and increases the secretion of several proteins into the supernatant.	^19,20^
	Ckf	Virulence factor	Paralogues of Ckf that disrupt host membranes when produced in excess.	^9^
OI-93	Stx1A Stx1B	Virulence factor	Stx1A and Stx1B are the 2 subunits of Shiga toxin 1. Shiga toxin inhibits host protein synthesis and increases EHEC O157:H7 survival in macrophages.	^16–18^
OI-122	PagC	Virulence factor	Paralogue of PagC in *Salmonella* increases bacterial survival in macrophages. Paralogue of PagC in *Citrobacter rodentium* promotes bacterial colonization in vivo.	^21–23^
	Efa1’	Virulence factor	Efa1ʹ promotes EHEC O157:H7 adherence to cultured HeLa cells and increases the expression and secretion of LEE-encoded proteins.	^24^
OI-148 (LEE PAI)	Esc Sep Esp	Virulence factor	Esc, Sep, and Esp proteins are components of the T3SS that allows the direct injection of bacterial effector proteins into host cells to subvert host cell signaling pathways and form AE lesions.	^25–28^
	Eae	Virulence factor	Eae, the intimin adhesion protein, interacts with its receptor Tir and mediates the intimate attachment of EHEC O157:H7 to host epithelial cells and the formation of A/E lesions.	^29^
O islands containing effectors				
OI-26	EspY3	Effector	EspY3 localizes in the pedestal region. EspY3 induces the elongation of polymerized actin pedestals in infected host epithelial cells, and generates a significant increase in the size of the pedestal areas.	^30^
OI-36	NleC	Effector	NleC acts as a zinc protease that suppresses the activation of NF-κB by directly cleaving the NF-κB subunit p65, and subsequently impairs the secretion of IL-1β, IL-8, and TNF-α.	^31,32^
	NleH1	Effector	NleH1 binds directly to a subunit of NF-kB, the ribosomal protein S3 (RPS3) to dampen host transcriptional outputs.	^33^
	NleD	Effector	NleD as a metalloprotease, directly cleaves p38 (a crucial part of the MAPK signaling pathway) and the p65 subunit of NF-κB to suppress the host immune responses.	^34,35^
OI-50	EspK	Effector	EspK localizes to the cytoplasm. EspK increases the persistence of EHEC O157:H7 in the intestine of orally-inoculated calves.	^36^
	NleL	Effector	NleL is an HECT-type E3 ligase, which modulates the formation of pedestals for adherence of EHEC O157:H7 to host epithelial cells. NleL-mediated monoubiquitination of c-Jun NH2-terminal kinases (JNKs) prevents their interaction with the upstream kinase MKK7, thus disrupting the phosphorylation and activation of the JNK pathway. NleL also disrupts host NF-κB signaling by targeting TRAF2, TRAF5, TRAF6, IKKα, and IKKβ.	^37–39^
OI-57	NleG2-3 NleG5-2 NleG6-2	Effector	NleG effectors, including NleG2-3, NleG5-2, and NleG6-2 are E3 ubiquitin ligases analogous to RING finger and U-box enzymes in eukaryotes. NleG2-3 resides in the host cytosol and triggers the ubiquitination-mediated degradation of hexokinase-2 and SNAP29. The exact functions of NleG2-3, NleG6-2, and NleG 5–2 in EHEC O157:H7 virulence and infection remain unclear.	^40,41^
OI-71	NleA	Effector	NleA localizes to the Golgi apparatus. NleA directly binding to Sec24 to compromise the Sec23/24 complex, which is a component of the mammalian COPII protein coat that shapes intracellular protein transport vesicles. NleA also directly targets the Nod-like receptor 3 (NLRP3) to reduce the formation of the NLRP3 inflammasome and prevent the activation of caspase-1 in host cells.	^42–44^
	NleF	Effector	NleF counteracts the host inflammatory response by dampening the caspase-4-mediated inflammatory epithelial cell death and preventing the production of IL-1β.	^45^
	NleH2	Effector	NleH2 binds directly to RPS3 and stimulates RPS3-dependent transcription, as well as an AP-1-dependent reporter.	^33^
	EspM1	Effector	EspM1 activates the RhoA pathway and induces the formation of stress fibers upon infection of host cells. In addition, EspM1 also represses the formation of actin pedestals during EHEC O157:H7 infection.	^46,47^
OI-79	EspJ	Effector	EspJ, as a unique ADP ribosyltransferase, directly inhibits Src kinase activity by simultaneous amidation and ADP ribosylation of the conserved kinase-domain residue, Src E310, resulting in glutamine-ADP ribose. EspJ also affects the dynamics of the clearance of *C. rodentium* from the intestinal tract of the host, suggesting the role for EspJ in host survival and pathogen transmission.	^48,49^
	EspF_U_	Effector	EspF_U_ interacts with the GTPase-binding domain (GBD) to activate N-WASP, recruiting the Arp2/3 complex and leading to actin polymerization. EspF_U_ also stabilizes the bacterial associations with the epithelial cytoskeleton and promotes the expansion of the infection beyond its initial sites.	^50,51^
OI-108	EspM2	Effector	EspM2 activates the RhoA pathway and induces the formation of stress fibers upon infection of host cells. In addition, EspM1 also represses the formation of actin pedestals during EHEC O157:H7 infection.	^46,47^
OI-122	EspL2	Effector	EspL2 targets annexin 2 in host cells, increases the bundling activity of F-actin and strengthens the membrane–cytoskeleton linkage.	^52^
	NleB1	Effector	NleB1 directly inactivates the death domains in several proteins (including TRADD, FADD, RIPK1, and TNFR1), and disrupts inflammatory NF-κB signaling, caspase 8-dependent apoptosis, and necroptosis.	^53,54^
	NleE	Effector	NleE inhibits the activation of NF-κB by preventing the activation of IKKβ and consequently the degradation of the IκB NF-kB inhibitor. NleE also inhibits the nuclear translocation of NF-κB subunit p65, thereby reducing the IL-8 response during bacterial infection.	^55,56^
OI-148 (LEE PAI)	Tir	Effector	Tir is translocated into the host cell membrane by T3SS and serves as a receptor for the intimin adhesion protein on the bacterial surface. Tir interacts with Eae and mediates the intimate attachment to host epithelial cells and formation of A/E lesions in EHEC O157:H7.	^26^
	Map	Effector	In initial stages of EHEC infection, Map is responsible for the transient formation of filopodium-like structures at the sites of bacterial infection. Map is also essential for the disruption of the function of the intestinal barrier and alteration of tight junctions.	^57,58^
	EspF	Effector	EspF is essential for the disruption of the function of intestinal barrier, being required for the loss of transepithelial resistance, for increased monolayer permeability, and for redistribution of the tight junction-associated protein occluding.	^59^
	EspG	Effector	EspG triggers the formation of actin stress fibers and destruction of microtubule networks underneath adherent bacteria in fibroblasts.	^60^
	EspH	Effector	EspH localizes to the host cell membrane and modulates the host actin cytoskeleton structure, affecting the formation of filopodium and pedestal sturctures	^61^
	EspB	Effector	EspB localizes to the region of bacterial attachment, and binds α-catenin, a cytoskeleton-associated molecule. Host cells transfected with EspB display altered morphology associated with a reduced number of stress fibers.	^62–64^
	EspZ	Effector	EspZ is involved in pedestal formation and localizes in pedestals alongside phosphorylated Tir. EspZ also acts as a gatekeeper to regulate the translocation of Tir, as well as other effectors including Map and EspF.	^65,66^
O islands containing regulatory proteins				
OI-9	OvrB	Regulatory protein	OvrB directly binds to the promoter region of LEE1 and activates the transcription of *ler*, which in turn activates LEE1–5 genes to promote the adherence of EHEC O157:H7 to host cells.	^67^
OI-19	OvrA	Regulatory protein	OvrA positively regulates the adherence of EHEC O157:H7 by activating the expression of LEE genes through the direct binding of OvrA to the *ler* gene promoter region.	^68^
OI-47	GrvA	Regulatory protein	GrvA positively regulates LEE by indirectly downregulating GadE, a regulator of acid tolerance and known repressor of *ler*.	^69,70^
OI-50	PsrA	Regulatory protein	PsrA indirectly represses type III secretion through the GadE and YhiF GAD acid stress response regulators.	^71^
OI-51	RgdR	Regulatory protein	RgdR activates transcription from the LEE1 promoter, leading to the induction of the Ler autoregulatory cascade that in turn promotes the expression of the remaining LEE operons and allows for type III secretion.	^72^
OI-57	PsrB	Regulatory protein	PsrB indirectly represses type III secretion through the GadE and YhiF GAD acid stress response regulators.	^71^
OI-115	EtrA	Regulatory protein	EtrA suppresses the expression of LEE genes to reduce type III secretion and adhesion to human intestinal cells.	^73^
	EivF	Regulatory protein	EivF suppresses the expression of LEE genes to reduce type III secretion and adhesion to human intestinal cells.	^73^
	EtrB	Regulatory protein	EtrB directly interacts with the ler regulatory region to activate the expression of LEE genes and promote the formation of AE lesions. EtrB activates the expression of LEE genes, not only through direct regulation but also by repressing the expression of *eivF* and *etrA*.	^74^
OI-119	LmiA	Regulatory protein	LmiA directly binds to the *ler* promoter region and activates the transcription of *ler*, which in turn activates LEE1–5 genes under low-magnesium conditions. The response of LmiA to a low-magnesium signal is mediated by the PhoQ/PhoP two-component system.	^75^
OI-148 (LEE PAI)	Ler	Regulatory protein	Ler binds to LEE promoters and activates the expression of all LEE operons. Ler is also required for the expression of non-LEE located virulence genes, including *espC, stcE*, and *lpf*.	^76–78^
	GrlA	Regulatory protein	GrlA binds to the LEE1 promoter and activates the expression of LEE genes through Ler.	^79,80^
	GrlR	Regulatory protein	GrlR directly binds to GrlA, preventing its interaction with the LEE1 promoter to repress the transcription of *ler*, which in turn represses the expression of other LEE genes.	^81,82^
O islands containing regulatory sRNAs				
OI-43	Esr41	Regulatory sRNA	Esr41 regulates the expression of *ler* at a post-transcriptional level in an Hfq-dependent manner; the mechanism of regulation remains unclear.	^83^
OI-93	Esr055	Regulatory sRNA	Esr055 represses the adherence of EHEC O157:H7 to HeLa cells. The expression of Esr055 is directly activated by DeoR; its expression is positively affected by DNA.	^84^
OI-148 (LEE PAI)	Arl	Regulatory sRNA	Arl post-transcriptionally regulates the *ler*-encoded LEE1 mRNA by specifically targeting the 3′ region of *ler*, preventing the translation of Ler, and thus the expression of LEE genes.	^85^
	sRNA350	Regulatory sRNA	sRNA350 promotes the expression of *ler, sepL, espA, tir, eae*, and *escV*.	^86^

**Figure:**
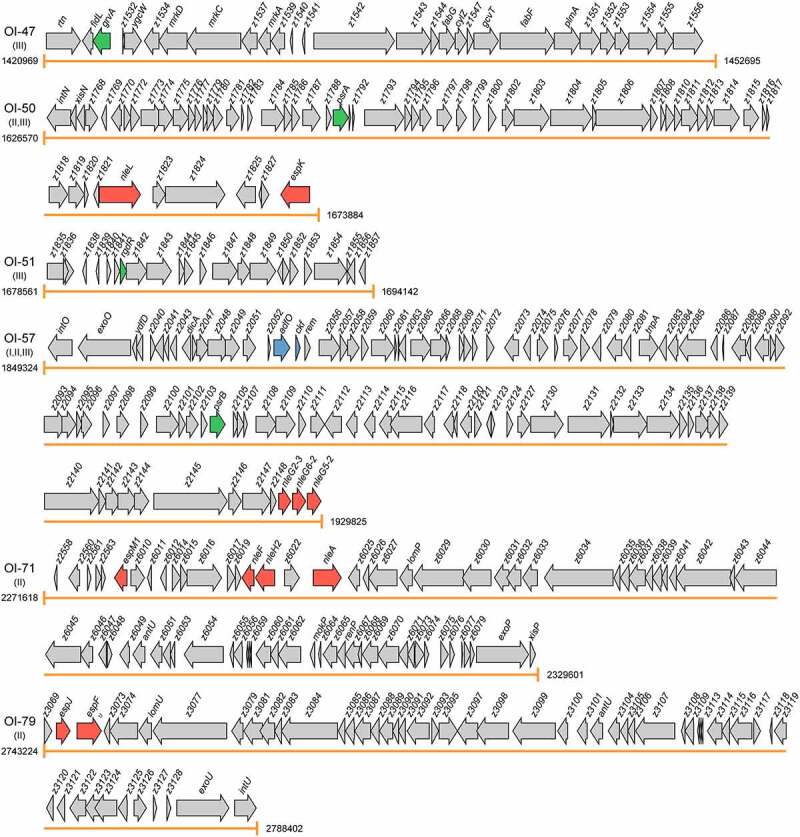

**Figure:**
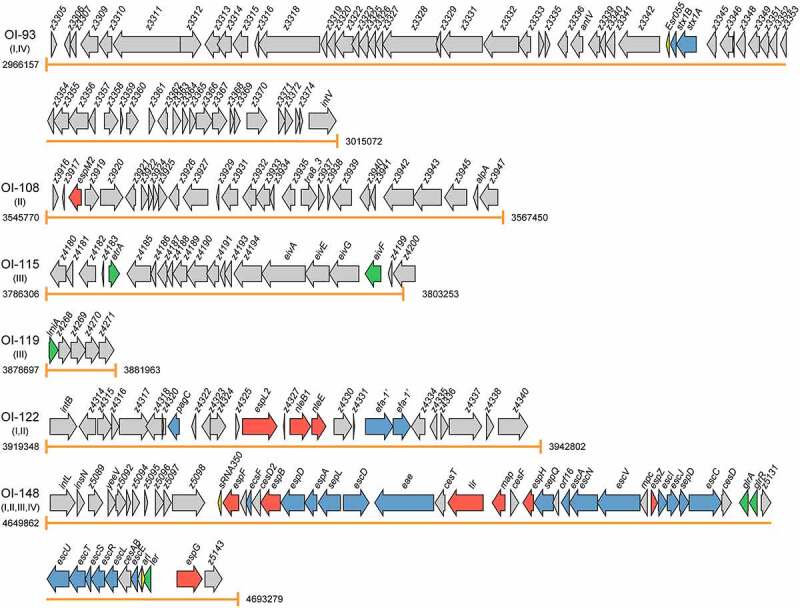

### OI-15

**Figure 3.**
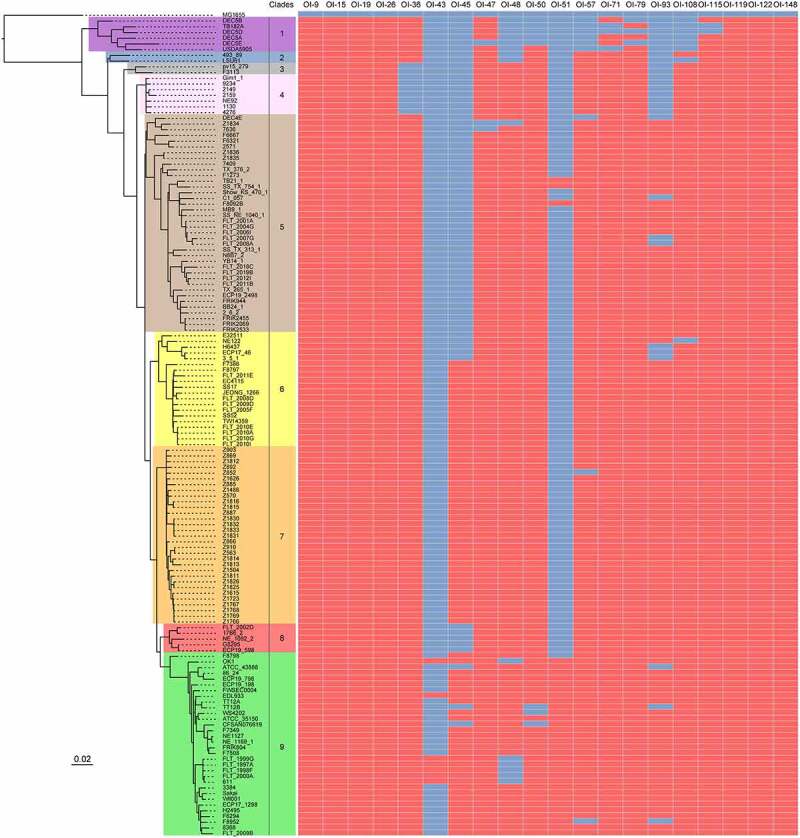
The prevalence of virulence-related OIs in EHEC O157:H7 strains. Maximum likelihood tree was constructed using PhyML based on 3440 single-copy core genes shared between *E. coli* K-12 MG1655 and 143 EHEC O157:H7 strains. Based on the phylogenetic analysis, these EHEC O157:H7 strains are classified into nine distinct clades (clade 1 to 9). The prevalence of different virulence-related OIs in EHEC O157:H7 strains was determined using BLASTN searches and are shown using heatmap. The search criteria are coverage ≥85% and identity ≥90%. Red and blue cells represent the presence and absence of OIs in a particular EHEC O157:H7 strain, respectively

OI-15 is a 4643 bp genomic region (ranging from 379203 bp to 383845 bp in the EHEC O157:H7 EDL933 genome). The phylogenetic analyses of complete genome sequences of 143 available EHEC O157:H7 strains revealed that these EHEC O157:H7 strains have been classified into 9 distinct clades (from clade 1 to clade 9, Figure 3). BLASTN searches showed that OI-15 is highly conserved and widely distributed in all 143 EHEC O157:H7 strains of 9 clades (Figure 3 and Table S1). OI-15 contains only a single open reading frame (ORF), *ehaA* (*z0402*).^9^ The *ehaA* gene encodes the AID15 adhesin, which contains a pertactin domain encompassing part of the repetitive beta-helical domain, followed by a C-terminal autotransporter domain encompassing the beta-barrel domain. Deletion of *ehaA* was reported to cause a significant reduction in the adherence of EHEC O157:H7 in pig ileal loops but not to HEp-2 and IPEC-J2 cells.^13^ Timothy and his colleagues demonstrated that EhaA is located at the cell surface, and its overexpression in *E. coli* K-12 cells resulted in the formation of large cell aggregates, promoted significant biofilm formation, and mediated adhesion to primary epithelial cells of the bovine terminal rectum.^14^

### OI-43/48

Contain a Pfam: Pertactin domain encompassing part of the repetitive beta-helical (passenger) domain, followed by a C-terminal Pfam: Autotransporter domain encompassing the beta-barrel (translocation) domain. contain a Pfam: Pertactin domain encompassing part of the repetitive beta-helical (passenger) domain, followed by a C-terminal Pfam: Autotransporter domain encompassing the beta-barrel (translocation) domain.

OI-43 is an 87563 bp island (ranging from 1058620 bp to 1146182 bp in the EHEC O157:H7 EDL933 genome) containing 97 ORFs (from *z1129* to *z1226*). OI-48 comprises an 87548 bp island (ranging from 1454242 bp to 1541789 bp according to the EHEC O157:H7 EDL933 genome) including 106 ORFs (from *z1559* to *z1664*). The EHEC O157:H7 strain EDL933 contains both the OI-43 and OI-48 islands, which are duplicates, whereas the other EHEC O157:H7 strains contain either OI-43 or OI-48 (Figure 3 and Table S1). In addition to the strain EDL933, six EHEC O157:H7 strains of clade 9 contain OI-43, including OK1, 611, FLT_1999 G, FLT_1997A, FLT_1998 F, and FLT_2000A (Figure 3 and Table S1). OI-48 is widely distributed in EHEC O157:H7 strains from clade 3 to clade 9, except for one strain in clade 5 (strain Z1834) and six strains that contain OI-43 in clade 9 (Figure 3 and Table S1). The genes in OI-43/48 are classified into 3 groups: a 7-gene cluster *ureDABCEFG*, which encodes urease and accessory proteins hydrolyzing *urea* to ammonia and carbon dioxide; telluride resistance genes *terZABCDEF*; and 2 putative adhesin genes, *iha* (encoding an IrgA homolog adhesin), and *aidA-1* (autotransporter adhesin involved in diffuse adherence).^10^ The telluride resistance proteins, Iha adhesin, and urease were shown to contribute to EHEC O157:H7 pathogenesis by promoting the adherence of the pathogen to the intestinal epithelium of the host. Deletion of the telluride resistance gene cluster reduced the ability of EHEC O157:H7 to adhere to and form large clusters on IPEC-J2 and HEp-2 cells,^15^ whereas, deletion of the *iha* and *ureC* genes had no effect on bacterial adherence in vitro but was found to significantly reduce the colonization of EHEC O157:H7 in ligated pig intestines. In contrast, deletion of the *aidA* gene had no effect on bacterial adherence neither in vitro nor in vivo.^15^

### OI-45/93

OI-45 and OI-93 are Stx-converting bacteriophages. Of note, Stxs are bacteriophage-encoded cytotoxins that damage a variety of cell types.^87^ Moreover, Stxs are divided into two groups, i.e., Stx1 and Stx2, with a 56% homology in their amino acid sequences. OI-45 is a 61663 bp island (ranging from 1330836 bp to 1392498 bp according to the EHEC O157:H7 EDL933 genome) that contains 69 ORFs (from *z1424* to *z1504*). OI-45 is present in all the EHEC O157:H7 strains of clade 7 and in the majority of strains in clade 9 (except for strains ATCC 43888, TT12B, and CFSAN076619) and clade 6 (except for strains E32511, NE122, H6437, ECP17-46, and 3-5-1) (Figure 3 and Table S1). In particular, *z1464* and *z1465* encode the A (Stx2A) and B (Stx2B) subunit of Stx 2, respectively. OI-93 is a 48916 bp island (ranging from 2966157 bp to 3015072 bp according to the EHEC O157:H7 EDL933 genome) that contains 65 ORFs (from *z3305* to *z3375*). OI-93 is widely distributed in EHEC O157:H7 strains from clade 5 to clade 9, except for four strains of clade 5 (strains DEC4E, C1_057, FLT_2007 G, and FLT_2006A), three strains of clade 6 (strains H6437, ECP17-46, and 3-5-1), and three strains of clade 9 (strains ATCC 43888, TT12B, and F8952) (Figure 3 and Table S1). Similarly, *z3344* and *z3343* encode the A (Stx1A) and B (Stx1B) subunit of Stx 1, respectively. It is known that Stxs play essential roles in the initial step of the colonization of the intestinal mucosa. After crossing the intestinal barrier, the B subunit of Stxs interacts with the globotriaosylceramide (Gb3) or globotetraosylceramide (Gb4) host receptors, allowing the internalization of the A subunit to the cytoplasm.^16^ The A subunit of Stxs inhibits the synthesis of host proteins through the specific removal of a single adenine residue from the 28S rRNA of the 60S ribosomal subunit.^17^ Stxs have also been implicated in the interaction between EHEC O157:H7 and human macrophages. Global gene expression profiling revealed that Stx genes were significantly upregulated in EHEC O157:H7 infected macrophages.^18^ Subsequent survival and cytotoxicity assays found that the initial uptake of Stx mutants was higher than that of wild-type; however, survival rates were significantly lower at 24 h postinfection.^18^

### OI-57

OI-57 is an 80502 bp island (ranging from 1849324 to 1929825 in the EHEC O157:H7 EDL933 genome) that contains 97 ORFs (from *z2048* to *z2151*). OI-57 is widely distributed in EHEC O157:H7 strains from clade 2 to clade 9, except for one strain of clade 5 (strain DEC4E), one strain of clade 7 (strain Z852), and one strain of clade 9 (strain F8952) (Figure 3 and Table S1). The *adfO* gene (*z2053*) encodes a virulence factor, which exhibits similarity to Paa, a virulence-associated protein of the porcine enteropathogenic *E. coli* (EPEC) that was implicated in the colonization of the pig ileum.^19^ An EHEC O157:H7 *adfO* mutant was reported to exhibit marked reductions in its ability to adhere to HeLa cells as well as to produce and secrete several proteins (including *z1931* and *z3065*) into the supernatant.^20^ The *adfO* virulence gene has been found to always be present together with *ckf* (*z2054*), which encodes a putative phage-associated bacterial cell-killing factor.^88^ When produced in excess, paralogues of Ckf have been reported to disrupt bacterial host membranes.^9^

### OI-122

OI-122 is a 23455 bp island (ranging from 3919348 bp to 3942802 bp in the EHEC O157:H7 EDL933 genome), that contains 26 ORFs (from *z4313* to *z4340*). OI-122 is widely distributed in all the 143 EHEC O157:H7 strains of 9 clades (Figure 3 and Table S1). In particular, *pagC* (*z4321*) encodes a virulence factor, which has significant homology to the *Salmonella enterica* serovar typhimurium PagC, that is important for bacterial survival and upregulated in macrophages.^21,22^ A *pagC* mutant in *Citrobacter rodentium*, which causes A/E lesions in mice, had a significantly lower competitive index, indicating its importance for the establishment of in vivo infection.^23^ Of note, Efa1 (encoded by *efa1*) was first identified as a factor influencing the adhesion of a clinical EHEC O111:H- strain to cultured epithelial cells.^89^ However, EHEC O157:H7 lacks the full-length *efa1* gene, but carries a truncated version of *efa1* (*efa1ʹ*) in the chromosome (*z4332* and *z4333*). Accordingly, a EHEC O157:H7 *efa1ʹ* mutant was shown to exhibit reduced adherence to cultured HeLa cells and reduced expression and secretion of LEE-encoded proteins.^24^ However, the *efa1ʹ* mutation did not significantly affect the course of fecal shedding of EHEC O157:H7 following experimental inoculation of 10- to 14-d-old calves or 6-week-old sheep.^24^

### OI-148

OI-148 is a 43418 bp island (ranging from 4649862 bp to 4693279 bp in the EHEC O157:H7 EDL933 genome) that contains 54 ORFs (from *z5087* to *z5143*). OI-148 is highly conserved and widely distributed in all the 143 EHEC O157:H7 strains of 9 clades (Figure 3 and Table S1). OI-148 harbors the LEE pathogenicity island, which is required for the intimate adherence of EHEC O157:H7 to host epithelial cells and the formation of A/E lesions. More specifically, LEE contains 41 genes (from *z5087* to *z5143*) grouped into 5 polycistronic operons (LEE1–LEE5).^7^ Among them, LEE1, LEE2, and LEE3 harbor the *esc* and *sep* genes, which encode for the components of the T3SS that allow the direct injection of bacterial effector proteins into host cells resulting in the subversion of host cell signaling pathways and formation of A/E lesions.^25^ The LEE5 operon contains *E. coli* attaching and effacing (*eae*) and *tir* genes, encoding for the intimin adhesion protein and its Tir receptor.^29^ The LEE4 contains the genes encoding translocator proteins, which form the tip of the T3SS (EspB and EspD), the EspA needle filament, the EscF needle protein, the CesD2 chaperone, the SepL gatekeeper protein, and the EspF effector protein.^26^ Deletion of LEE has been demonstrated to completely abolish the ability of EHEC O157:H7 to adhere to host cells and form A/E lesions.^27^ Intriguingly, the EPEC LEE expressed from a multicopy plasmid transformed into an *E. coli* K-12 laboratory strain was necessary and sufficient to give rise to the A/E phenotype on human epithelial cells in culture.^90^ In contrast, the EHEC O157:H7 LEE alone was not sufficient to confer the A/E phenotype when expressed in an *E. coli* laboratory strain,^28^ suggesting that certain factors or regulatory proteins outside of EHEC LEE are necessary for the A/E phenotype.

## OIs encoding effectors

The effectors of EHEC O157:H7 are encoded on either the LEE, prophages, or insertion elements.^91^ These effectors are known to be key modulators of the innate immune system of intestinal epithelial cells, and have been found to exert their function mainly through disturbing the nuclear factor kappa Β (NF-κB)-regulated signal transduction pathways.^92,93^ Once translocated, these effectors are targeted to various intracellular compartments and modulate diverse signaling pathways and physiological processes, including ion secretion, apoptosis, membrane insertion, and cytoskeleton changes.^94^ To date, eight OIs, namely OI-26, OI-36, OI-50, OI-57, OI-71, OI-79, OI-122, and OI-148 have been reported to harbor genes encoding effector proteins (Figure 2 and Table 1).

### OI-26

OI-26 is a 1983 bp region (ranging from 501358 bp to 503340 bp in the EHEC O157:H7 EDL933 genome), which is highly conserved and widely distributed in all the 143 EHEC O157:H7 strains of 9 clades (Figure 3 and Table S1). OI-26 contains only a single ORF, i.e., *espY3* (*z0521*). The EspY3 protein contains an N-terminal WEX5F domain with homology to SopD (*Salmonella* outer protein D), a well-characterized T3SS-1 (SPI-1) effector protein of *Salmonella*.^95^ EspY3 has been shown to localize in the pedestal region.^30^ The EspY3 effector was reported to induce the elongation of polymerized actin pedestals in infected host epithelial cells, thus inducing a significant increase in the size of pedestal areas.^30^

### OI-36

OI-36 is a 38588 bp island (ranging from 892772 bp to 931359 bp in the EHEC O157:H7 EDL933 genome) that contains 42 ORFs (from *z0946* to *z0990*). Bioinformatics analysis showed that OI-36 existed in EHEC O157:H7 strains of seven clades (clades 1, 2, and 5 to 9) but not in the strains of the other two clades (clades 3 and 4) (Figure 3 and Table S1). The *nleC* (*z0986), nleH1* (*z0989*), and *nleD* (*z0990*) genes within OI-36 encode three translocated effectors.^96^ Particularly, NleC, which acts as a zinc protease, was found to compromise the activation of NF-κB by directly cleaving the NF-κB subunit p65,^31^ resulting in a decrease in the total nuclear entry of active p65. Consequently, NleC-mediated proteolysis suppressed the activation of NF-κB and in turn impaired the secretion of interleukin-1β (IL-1β), IL-8, and tumor necrosis factor-α (TNF-α).^31,32^ NleH1 has significant sequence similarity to *Shigella flexneri* OspG, a protein known to interfere with the activation of NF-kB^97^ and has been shown to bind directly to a subunit of NF-kB, the ribosomal protein S3 (RPS3).^33^ One of the functions of RPS3 is to guide the recruitment of the p65 NF-κB subunit to specific promoters in response to different stimuli. NleH1 functions by reducing the nuclear abundance of RPS3 to dampen host transcriptional outputs.^33^ Furthermore, deleting *nleH1* from EHEC O157:H7 produced a hypervirulent Stx-producing *E. coli* phenotype in a gnotobiotic piglet model.^33^ Finally, NleD is a metalloprotease, which directly cleaves p38, a crucial part of the MAPK signaling pathway, and JNK within the activation loop in pathogen-infected epithelial cells, thus suppressing the host immune response.^34^ In addition, NleD was also shown to specifically cleave and inactivate the p65 subunit of NF-κB.^35^

### OI-50

OI-50 is a 47315 bp island (ranging from 1626570 bp to 1673884 bp in the EHEC O157:H7 EDL933 genome) that contains 162 ORFs (from *z1664* to *z1829*). OI-50 is widely distributed in EHEC O157:H7 strains from clade 2 to 9, except for three strains of clade 9 (strains TT12B, WS4202, and CFSAN076619) (Figure 3 and Table S1). The *espK* (*z1829), espN* (*z1824*), and *nleL* (*z1822*) genes within OI-50 encode translocated effectors. Interestingly, EspK is homologous to the Gifsy phage-encoded *Salmonella enterica* serovar typhimurium type III secreted effector GogB.^98^ When transiently expressed in COS-7, a cell line of immortalized kidney fibroblasts from the African green monkey, EspK localizes to the cytoplasm.^36^ Inactivation of *espK* did not impair the adherence or actin nucleation during the infection of HeLa cells but significantly reduced the persistence of EHEC O157:H7 in the intestine of orally inoculated calves.^36^ NleL (previously known as EspX7) is an HECT-type E3 ligase, which modulates the formation of the pedestal used for the adherence of EHEC O157:H7 to host epithelial cells.^37,38^ The c-Jun NH2-terminal kinases (JNKs) are known to constitute host substrates of NleL, and their NleL-mediated monoubiquitination has been shown to prevent their interaction with the upstream kinase MKK7, thus disrupting the phosphorylation and activation of the JNK signaling pathway.^38^ A recent work revealed that NleL disrupted the host NF-κB signaling by targeting several components of the NF-κB pathway, including TRAF2, TRAF5, TRAF6, IKKα, and IKKβ.^39^

### OI-57

The NleG homologs constitute the largest family of T3SS-delivered effectors, with 14 members in EHEC O157:H7. In addition to encoding for two virulence factors (AdfO and Ckf), OI-57 also encodes three NleG effectors, i.e., NleG2-3 (*z2149*), NleG6-2 (*z2150*), and NleG5-2 (*z2151*).^88^ The C-terminal domain of NleG2-3 (residues 90 to 191) is the most conserved region in NleG proteins, containing a RING finger/U-box motif.^40^ Bacterial-encoded NleG effectors, including NleG2-3, NleG6-2, and NleG5-2 were demonstrated to function as E3 ubiquitin ligases analogous to RING finger and U-box enzymes in eukaryotes.^40^ The NleG2-3 effector was found to reside in the host cytosol, triggering the ubiquitination-mediated degradation of hexokinase-2 and SNAP29 (synaptosomal-associated protein of 29 kDa).^41^ However, the exact functions of NleG2-3, NleG6-2, and NleG 5–2 in EHEC O157:H7 virulence and infection remain unclear.

### OI-71

OI-71 is a 57984 bp island (ranging from 2271618 bp to 2329601 bp in the EHEC O157:H7 EDL933 genome) that contains 77 ORFs (including *z2558, z2560, z2561, z2562, z2563, z2565*, and *z6010* to *z6081*). OI-71 is widely distributed in EHEC O157:H7 strains from clade 1 to 9, except for three strains of clade 1 (strains TB182A, DEC5D, and USDA5905) (Figure 3 and Table S1). OI-71 contains four effector genes, including *nleA* (*z6024*, also called *espI), nleF* (*z6020), nleH2* (*z6021*), and *espM1* (*z2565*).^9^ The NleA effector localizes to the Golgi apparatus.^42^ NleA was first shown to compromise the Sec23/24 complex, which is a component of the mammalian COPII protein coat that shapes intracellular protein transport vesicles by directly binding to Sec24.^43^ Moreover, NleA is known to directly target the Nod-like receptor 3 (NLRP3), one of the three basic components of the inflammasome; in particular, NleA interrupts the deubiquitination of NLRP3, which is a prerequisite for the assembly of the inflammasome.^44^ Consequently, NleA reduces the formation of the NLRP3 inflammasome and prevents the activation of caspase-1 in host cells.^44^ Similarly, NleF localizes to the cytoplasm.^99^ Challenge of gnotobiotic piglets with wild-type and *nleF* mutant EHEC O157:H7 revealed a role of NleF in the colonization of the colon and rectoanal junction of piglets.^99^ EHEC O157:H7 was also reported to use NleF to counteract the host inflammatory response by dampening the caspase-4-mediated inflammatory epithelial cell death and preventing the production of IL-1β.^45^ In addition, NleF not only interrupted the heterodimerization of caspase-4-p19 and caspase-4-p10, but also inhibited the interaction of caspase-1 and caspase-4.^45^ NleH2 (303 amino acids) shares an 84% amino acid sequence identity with NleH1 (293 amino acids). Similar to NleH1, NleH2 also binds directly to RPS3, and colocalized with RPS3 in the cytoplasm, but not in cell nuclei.^33^ In contrast to NleH1, NleH2 stimulated the RPS3-dependent transcription, as well as an AP-1-dependent reporter.^33^ EHEC O157:H7 encodes two *espM* alleles, *espM1* and *espM2*, which reside in OI-71 and OI-108, respectively. Both EspM1 and EspM2 effectors were found to activate the RhoA pathway and induce the formation of stress fibers upon infection of host cells.^46^ In addition to the induction of the formation of stress fibers, both EspM1 and EspM2 also repressed the formation of actin pedestals during an EHEC O157:H7 infection.^47^

### OI-79

OI-79 is a 45179 bp island (ranging from 2743224 bp to 2788402 bp in the EHEC O157:H7 EDL933 genome) that contains 54 ORFs (from *z3069* to *z3130*). OI-79 is widely distributed in EHEC O157:H7 strains from clade 1 to 9, except for three strains of clade 1 (strain DEC5B, DEC5D, and DEC5E) (Figure 3 and Table S1). The *espJ* (*z3071*) and *espFu* (*z3072*) genes encode translocated effectors. EspJ has an 22% sequence identity to HopF, an effector protein of *Pseudomonas syringae*.^100^ In EPEC, EspJ has been identified as a unique ADP ribosyltransferase that directly inhibits Src kinase activity by simultaneous amidation and ADP ribosylation of the conserved kinase-domain residue, Src E310, resulting in glutamine-ADP ribose.^48^ Although EspJ is not required for the formation of A/E lesions in HEp-2 cells or human intestinal explants, in-vivo studies performed using C57BL/6 J mice have shown that EspJ affects the dynamics of the clearance of *C. rodentium* from the intestinal tract of the host, suggesting the role for EspJ in host survival and pathogen transmission.^49^ EspF_U_ bridges the interaction of Tir with the host IRSp53 or IRTKS proteins and N-WASP.^50^ The C terminus of EspF_U_ then interacts with the GTPase-binding domain (GBD) to activate N-WASP, recruiting the Arp2/3 complex and leading to actin polymerization.^50^ After initial EHEC O157:H7 colonization of the intestinal surface, EspF_U_ was also demonstrated to stabilize the bacterial association with the epithelial cytoskeleton and promote the expansion of infection beyond its initial sites.^51^

### OI-122

OI-122, in addition to encoding virulence factors (*pagC* and *efa1*), encodes three effectors, i.e., EspL2 (*z4326*), NleB1 (*z4328*), and NleE (*z4329*).^101^ EspL2 localizes to the cytosolic side of the plasma membrane.^52^ Annexin 2 (also known as annexin II) is the target of EspL2 in host cells.^52^ The interaction of EspL2 with annexin 2 was shown to increase the bundling activity of F-actin and strengthen the membrane–cytoskeleton linkage, resulting in the condensation of actin fibers and the induction of a pseudopod-like structure.^52^ EHEC NleB1 has an 89% sequence identity to NleB from *C. rodentium*, with only a difference of 7 amino acids between the EHEC and EPEC polypeptides.^102^ NleB1 was reported to directly inactivate the death domains in several proteins (including TRADD, FADD, RIPK1, and TNFR1) dependent on its N-GlcNAc transferase activity.^53^ These modifications blocked the interactions between the death domains, thereby disrupting the inflammatory NF-κB signaling, caspase 8-dependent apoptosis, and necroptosis.^54^ NleE is a highly conserved T3SS effector protein of A/E pathogens that is encoded in the same operon containing NleB1. NleE inhibits the activation of NF-κB by preventing the activation of IKKβ and consequently the degradation of the IκB NF-kB inhibitor.^55^ This activity of NleE was shown to be enhanced by NleB1.^55^ In addition, NleE is also known to inhibit the nuclear translocation of the NF-κB subunit p65, thereby reducing the IL-8 response during bacterial infection.^56^

### OI-148

In addition to genes encoding virulence factors, seven genes (including *map, espF, espG, espH, espB, tir*, and *espZ*) encoding effectors are also present in OI-148.^103^ In initial stages of EHEC infection, the mitochondrial-associated Map protein was found to be responsible for the transient formation of filopodium-like structures at the sites of bacterial infection, with the process being dependent on the Cdc42 small G protein.^57^ Map is also essential for the disruption of the intestinal barrier function and alteration of tight junctions, and this activity was reported to be independent of mitochondrial targeting.^58^ EspF is a proline-rich effector protein in EHEC O157:H7, containing four proline-rich repeats.^104^ It has been shown to play a role in the disruption of the intestinal barrier function, being required for the loss of transepithelial resistance for increased monolayer permeability, and for the redistribution of the tight junction-associated protein occluding.^59^ EspG was also reported to trigger the formation of actin stress fibers and the destruction of microtubule networks underneath adherent bacteria in fibroblasts.^60^ It has also been shown to interact with tubulins, stimulating the destabilization of microtubules in vitro, with this destabilization triggering the activation of the RhoA-ROCK signaling pathway through the activation of the guanine nucleotide exchange factor (GEF-H1).^60^ EspH localizes to the host cell membrane and modulates the host actin cytoskeleton structure, affecting the formation of filopodium and pedestal structures.^61^ In addition to its role in translocation, EspB was reported to exert an effector activity. Cytosolic EspB was shown to localize to the region of the bacterial attachment,^62^ and cells transfected with EspB displayed altered morphology associated with a reduced number of stress fibers.^63^ Additionally, EHEC EspB has been shown to bind α-catenin, a cytoskeleton-associated molecule, consistent with its role in modulating the host cell cytoskeleton.^64^ Tir (also called EspE) serves as a receptor for the intimin adhesion protein on the bacterial surface, triggering a number of signaling cascades and leading to the formation of pedestal structures and A/E lesions.^26^ EspZ (also known as SepZ) has been shown to be localized in pedestals alongside phosphorylated Tir.^65^ The translocation of EspZ was demonstrated to be restricted to the formation of pedestals and not involved in the disruption of the integrity of cellular tight junctions or in mediating cytoskeletal rearrangements.^65^ EspZ has also been identified as a gatekeeper that regulates the translocation of Tir, as well as other effectors including Map and EspF.^66^

## OIs encoding virulence regulatory proteins

**Figure 4.**
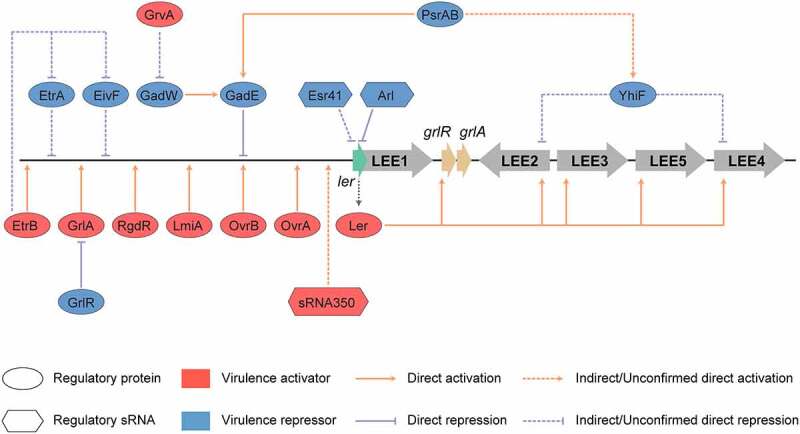
Regulation of LEE by regulatory proteins and sRNAs encoded in O islands. The master LEE regulator, Ler recognizes AT rich sequences and activates the transcription of LEE operons LEE2, LEE3, LEE4, and LEE5. The OvrA, OvrB, LmiA, RgdR, GrlA, and EtrB regulatory proteins encoded in O islands, directly activate the transcription of LEE genes through directly binding to the promoter region of LEE1. GrvA indirectly activates the expression of LEE genes through GadW and GadE. In contrast, GadE directly represses the expression of LEE genes, while EtrA, EivF, and YhiF indirectly repress the expression of LEE genes. PsrA and PsrB indirectly regulate LEE genes through the GAD acid stress response regulators, GadE and YhiF. The cis-encoded sRNA Arl regulates *ler* mRNA post-transcriptionally by specifically targeting its 3′ region, preventing its translation, and thus the expression of LEE genes. The Esr41 sRNA regulates the expression of *ler* at a post-transcriptional level in an Hfq-dependent manner; however, the mechanism of this regulation remains unclear. sRNA350 positively regulates the expression of LEE genes through a still unknown mechanism. The figure has been inspired by the previous work of Furniss et al. (2017), Bhatt et al. (2016), and Platenkamp et al. (2018)

To ensure the successful EHEC O157:H7 colonization, virulence-related genes are subjected to strict regulation that ensures their expression only under optimal environmental conditions, while also avoiding intense metabolic cost or alerting the host immune system.^76^ The coordinated expression of virulence genes is controlled by regulatory proteins, which precisely activate or repress particular genes depending on environmental factors and the stage of infection.^105^ Several OI-encoded virulence regulatory proteins have been demonstrated to affect the expression of EHEC O157:H7 virulence genes, and the precise molecular mechanisms underlying this regulation have also been revealed in recent years. These OIs encoding virulence regulatory proteins include OI-9, OI-19, OI-47, OI-50, OI-51, OI-115, OI-119, and OI-148 (Figures 2, 4, and Table 1).

### OI-9

OI-9 is a 6133 bp island (ranging from 323996 bp to 330128 bp in the EHEC O157:H7 EDL933 genome), which is highly conserved and widely distributed in all 143 EHEC O157:H7 strains of 9 clades (Figure 3 and Table S1). OI-9 comprises seven ORFs (from *z0342* to *z0348*). In particular, *z0342*, and *z0346* (also termed OI-encoded virulence regulator B, *ovrB*) are predicted to encode two putative regulators; *z0343* encodes an oxidoreductase, and *z0347* encodes a hydrolase; *z0348* encodes a major facilitator superfamily (MFS) transporter, whereas *z0344* and *z0345* encode hypothetical proteins of unknown function. We recently found that *ovrB* encodes a novel transcriptional activator, which is required for bacterial adherence to host cells and the expression of LEE genes in EHEC O157:H7.^67^ Deletion of *ovrB* significantly reduced bacterial adherence to HeLa and Caco-2 cells, as well as the expression of LEE genes in EHEC O157:H7. OvrB was demonstrated to directly bind to the promoter region of LEE1, activating the transcription of *ler* (encoding a master regulator of LEE genes), which in turn activates LEE1-5 genes to promote EHEC O157:H7 adherence. Electrophoretic mobility shift assay (EMSA) and chromatin immunoprecipitation quantitative PCR (ChIP-qPCR) analysis showed that OvrB directly bound to the LEE1 promoter, regulating the expression of LEE1-LEE5 via the master LEE regulator, Ler. Furthermore, mouse oral infection assays revealed that OvrB promoted EHEC O157:H7 colonization in the mouse intestine. We also showed that OvrB is a widespread transcriptional activator of virulence genes in different *E. coli* pathotypes among various pathogens. In contrast, deletion of other OI-9 genes (*z0342, z0343, z0344, z0345, z0347*, and *z0348*) had no visible effect on EHEC O157:H7 virulence, as both the adherence and levels of expression of LEE genes in all mutants were comparable to those of the wild-type EHEC O157:H7.^67^ We thus proposed that these genes might be redundant or involved in other bacterial processes. This hypothesis needs to be further investigated.

### OI-19

OI-19 is a 1834 bp island (ranging from 424359 bp to 426192 bp in the EHEC O157:H7 EDL933 genome), which is highly conserved and widely distributed in all the 143 EHEC O157:H7 strains of 9 clades (Figure 3 and Table S1). OI-19 includes two ORFs, *z0442* (also termed OI-encoded virulence regulator A, *ovrA*) and *z0443*. The *ovrA* gene has been predicted to encode a putative transcriptional regulator containing an AraC-like DNA-binding helix-turn-helix (HTH) domain (pfam 12833) in the C-terminal region, whereas *z0443* encodes a hypothetical protein of unknown function. We found that OvrA constitutes a novel transcriptional regulator that promotes bacterial adherence by activating the expression of LEE genes in EHEC O157:H7.^68^ Deletion of *ovrA* resulted in the reduced adherence of EHEC O157:H7 to cultured host cells (HeLa and Caco-2), and reductions in the formation of A/E lesions and expression of LEE genes. Conversely, *z0443* deletion did not obviously affect EHEC O157:H7 virulence. EMSA, ChIP-qPCR, and DNase I footprinting analyses revealed that OvrA directly bound to a 17-base pair motif (5′-GACATTTAATGATAATG-3′; −183 to −167 from the *ler* translational start site) within the LEE1 promoter region to activate the expression of *ler*, and in turn activate the expression of LEE1–LEE5 via the master LEE regulator Ler. Mouse colonization experiments revealed that OvrA promoted EHEC O157:H7 adherence in the mouse intestine, preferentially the colon. Moreover, OvrA was also shown to be involved in the regulation of virulence in other non-O157 pathogenic *E. coli*, including EHEC strains O145:H28 and O157:H16 and EPEC strain O55:H7.^68^

### OI-47

OI-47 is a 31727 bp island (ranging from 1420969 bp to 1452695 bp in the EHEC O157:H7 EDL933 genome) that encodes 27 ORFs (from *z1528* to *z1556*). OI-47 is widely distributed in nearly all EHEC O157:H7 strains of nine clades, except for one strain of clade 1 (strain DEC5E) and two strains of clade 5 (strains Z1834 and 7636) (Figure 3 and Table S1). GrvA (for global regulator of virulence A, *z1531*) encodes a Tox-R family transcriptional regulator, which has been found to be also involved in the regulation of LEE.^69^ Moreover, GrvA works in conjunction with RcsB, a regulator involved in the activation and repression of LEE; however, the underlying mechanism was not understood until recently.^69,70^ RcsB positively regulates the expression of *grvA* by directly binding to the *grvA* promoter region; this binding however is temperature sensitive.^70^ In addition, GrvA positively regulates LEE by indirectly downregulating GadE, a regulator of acid tolerance and known repressor of *ler*.^70,106^ Moreover, the repression of *gadE* by GrvA was reported to be dependent on an intact *gadW*.^70^

### OI-50

OI-50 also encodes a putative AraC/XylS family regulator, PsrA (prophage-encoded secretion regulator A, *z1789*). Deletion of *psrA* was shown to significantly increase T3SS secretion in a manner analogous to the deletion of the entire OI-50 region.^71^ Interestingly, PsrB (*z2104*), present on OI-57, is the closest homolog of PsrA (90% identity). Deletion of *psrB* did not have a significant effect on T3SS secretion but repressed secretion when provided in trans in the *psrA* deletion mutant. Both PsrA and PsrB were shown to indirectly regulate T3SS through the GAD acid stress response regulators GadE and YhiF. Furthermore, the same study also demonstrated that OI-50 was required for the persistence of infection in a ruminant model of colonization and that the effector encoding loci and PsrA regulator both contributed to this persistence phenotype.^71^

### OI-51

OI-51 is a 15582 bp island (ranging from 1678561 bp to 1694142 bp in the EHEC O157:H7 EDL933 genome) that contains 22 ORFs (from *z1835* to *z1857*). OI-51 existed in all the EHEC O157:H7 strains of clade 9 and three strains of clade 5 (strains TB21-1, SS TX 754–1, and F8092B) (Figure 3 and Table S1). Deletion of OI-51 resulted in a reduction in the expression of LEE and T3SS. In addition, the deletion led to a reduced capacity for attachment to epithelial cells, also significantly reducing the levels of EHEC O157:H7 excretion in sheep.^72^ Through a combination of deletion and complementation analyses, RgdR was identified as a novel regulator of OI-51, thereby able to stimulate T3SS and bacterial adherence. Mechanistically, the stimulation of T3SS by RgdR was reported to occur through the initial activation of transcription from the LEE1 promoter, leading to the induction of the Ler autoregulatory cascade that in turn promoted the expression of the remaining LEE operons and T3SS.^72^

### OI-115

OI-115 is a 16948 bp island (ranging from 3786306 bp to 3803253 bp in the EHEC O157:H7 EDL933 genome) that contains 21 ORFs (from *z4180* to *z4200*). OI-115 is widely distributed in nearly all EHEC O157:H7 strains of nine clades, except for two strains of clade 1 (strains TB182A and DEC5D) (Figure 3 and Table S1). OI-115 harbors a gene cluster encoding components of a second cryptic T3SS, *E. coli* type III secretion system 2 (ETT2). The functionality and role of the ETT2 system have been controversial, with the locus being subject to widespread mutational attrition.^107^ However, two system-encoded regulators, EtrA (*z4184*) and EivF (*z4198*), were found to be functional repressors of LEE in EHEC O157:H7.^73^ Mutational inhibition of *etrA* and *eivF* from the ETT2 cluster was demonstrated to lead to the significantly increased secretion of LEE-encoded proteins (including EspA, EspB, Tir, and EspP) and to increased adhesion to human intestinal cells (Int407). Transcriptional fusions and microarrays analyses indicated that EtrA and EivF exert profound negative effects on gene transcription within LEE. Consistent with these observations, the expression of these regulators in an EHEC O26:H- strain led to the suppression of protein secretion under LEE-inducing conditions.^73^ In addition to EtrA and EivF, ETT2 also encodes a third virulence regulator, EtrB (*z4167*), which was reported to play an important role in EHEC O157:H7 pathogenesis.^74^ The *etrB* gene is expressed as a monocistronic transcript, and EtrB positively autoregulates its expression. EtrB directly interacts with the *ler* regulatory region to activate the expression of LEE and promote the formation of A/E lesions. In particular, EtrB activates the expression of LEE, not only through direct regulation but also by repressing the expression of *eivF* and *etrA*. Moreover, EtrB was also found to modulate the expression of genes encoding products with distinct functions, including the non-LEE-encoded effector NleA, a fimbrial adhesin (Loc11), a small RNA (RyeA/SraC), and a gene involved in maltose and tryptophan metabolism.^74^

### OI-119

OI-119 is a 3267 bp island (ranging from 3878697 bp to 3881963 bp in the EHEC O157:H7 EDL933 genome), which is highly conserved and widely distributed in all the 143 EHEC O157:H7 strains of 9 clades (Figure 3 and Table S1). OI-119 contains five ORFs (from *z4267* to *z4271*). The z4267 gene (also termed *lmiA* for low-magnesium-induced regulator A) encodes a putative DNA-binding protein, *z4268* and *z4269* encode hypothetical proteins of unknown function, whereas *z4270* and *z4271* encode putative ATP-binding proteins of the ABC transport system. We recently discovered that LmiA, as a novel virulence regulator within OI-119, promoted the bacterial adherence to epithelial cells and expression of LEE genes, facilitating EHEC O157:H7 colonization.^75^ In contrast, a *ΔlmiA* mutant exhibited significantly reduced bacterial adherence, as evidenced by bacterial adherence and FAS assays, as well as suppressed the transcriptional and translational expression of LEE genes compared with that of the wild-type. EMSA, ChIP-qPCR, and DNase I footprinting analyses revealed that LmiA directly bound to a 17-base pair motif (5′- TTAAAGTCGTTTGTTAA −3′; −247 to −231 from the *ler* proximal transcriptional start site) within the LEE1 promoter region to activate the expression of *ler*, and in turn promote the expression of LEE1-5 genes through Ler. Furthermore, LmiA was reported to be an essential element that integrates the low-magnesium signals from the large intestine into this LEE regulatory network via the PhoP/PhoQ two-component regulatory system.^75^ This LmiA-mediated virulence regulatory pathway is widely present in a range of EHEC and EPEC serotypes. Disruption of this pathway significantly decreased EHEC O157:H7 adherence in the mouse intestinal tract. Moreover, feeding mice a magnesium-rich diet significantly reduced EHEC O157:H7 adherence in vivo. Therefore, our findings supported the use of magnesium as a dietary supplement and provided greater insights into the dietary cues that can prevent EHEC and EPEC infections in humans.^75^

### OI-148

OI-148 encodes three virulence regulatory proteins: Ler (locus of enterocyte effacement regulator), GrlA (global regulator of LEE activator), and GlrR (global regulator of LEE repressor). Ler, encoded by the first gene in the LEE1 operon, is an HNS-like regulatory protein^108^ that recognizes AT rich sequences and acts as a derepressor for the transcriptional silencing exerted by the histone-like nucleoid-structuring protein (H-NS). Ler is required for the increased transcription of LEE operons, LEE2, LEE3, LEE4, LEE5, as well as that of *grlRA* and *espF, espG*, and *map*.^77^ Additionally, Ler is also required for the expression of non-LEE located virulence genes, including espC, stcE, and lpf.^76,78^ Consistently, EHEC O157:H7 nonpolar *ler* mutants were unable to form A/E lesions on HEp-2 cells.^109^ In addition, these *ler* mutants failed to express type III secreted effectors and showed a decreased expression of non-LEE-encoded virulence factors.^109^ Both GrlA and GrlR are encoded by the *grlRA* operon located between the *rorf3* gene and the LEE2 operon in LEE. GrlA drives the expression of virulence genes, probably through its direct binding to the suboptimal 18-base-pair spacer between the −10 and −35 elements of the distal LEE1 promoter, P1, via a HTH DNA-binding motif.^79,80^ This binding has been suggested to drive the expression of *ler* and subsequently all the LEE genes. Conversely GrlR, the cellular levels of which are regulated in a growth phase-dependent manner,^81^ was shown to antagonize this system by directly binding to GrlA, preventing its interaction with the LEE1 promoter to repress the transcription of LEE genes.^82^

## OIs encoding virulence-sRNA

Small regulatory RNAs (sRNAs) are typically noncoding RNAs that are 50 to 300 nucleotides in length.^110^ The majority of sRNAs are known to target mRNAs, affecting the transcriptional elongation, stability, or translation of mRNAs.^110^ Moreover, sRNAs have been demonstrated to coregulate numerous biological processes, including oxidative stress, acid stress, motility, quorum sensing, antibiotic resistance, and virulence.^110^ A previous study reported that the density of sRNAs encoded in OIs was 39 sRNAs per Mb of DNA, compared with 23 sRNAs per Mb of DNA in the core genome.^111^ To date, four sRNAs encoded in OIs have been identified to be involved in the regulation of EHEC O157:H7 virulence, including Arl (OI-148), sRNA350 (OI-148), Esr41 (OI-43), and Esr055 (OI-93) (Figures 2, 4, and Table 1).

### OI-148

ggsOI-148 encodes two virulence regulatory sRNAs: Arl (antisense regulator of *ler* RNA) and sRNA350. The cis-encoded sRNA Arl was shown to influence the expression of LEE in EHEC O157:H7.^85^ Arl is located downstream of *ler* but transcribed from the antisense strand. Consequently, Arl exhibits extensive complementarity to the LEE1-encoded ler mRNA. The transcription of *arl* is stimulated by elevated cytoplasmic levels of iron or hydroxyl radical but does not require the iron-responsive transcriptional factor Fur.^85^ In addition, Arl regulates the *ler*-encoded LEE1 mRNA post-transcriptionally by specifically targeting the 3′ region of *ler*, preventing its translation, and thus the expression of LEE genes.^85^ sRNA350 is located downstream of the *cesF* gene, and acts as a global regulator of the LEE island.^86^ Overexpression of sRNA350 in wild-type EHEC O157:H7 resulted in the upregulation of various genes within LEE, including *ler, sepL, espA, tir, eae*, and *escV*, as measured by qRT-PCR.^86^ However, the transcript levels of *nleA* were unaffected.^86^

### OI-43

The OI-43 encoded sRNA Esr41 (EHEC O157 small RNA #41) is located in an intergenic region between *z1181* and *z1182*. Esr41 is approximately 70 nucleotides long and harbors a 3ʹ GC-rich palindrome sequence followed by a long poly(U), which is characteristic of rho-independent terminators and also a structural feature required for the action of Hfq.^112^ Esr41 was reported to repress the expression of LEE and reduce EHEC O157:H7 adhesion to host cells.^83^ EHEC O157:H7 harboring an *esr41-*expressing multicopy plasmid abolished the expression of LEE by downregulating the expression of *ler* and *pch* that are known positive regulators of LEE.^83^ Esr41 regulates the expression of *ler* at a post-transcriptional level in an Hfq-dependent manner.^83^ However, while the repression of LEE appears to be attributed to an interaction between the leader region of the *ler* mRNA and Esr41, no sites of complementarity have been identified between Esr41 and *ler*, and hence the mechanism of regulation remains unclear.

### OI-93

The OI-93 encoded sRNA Esr055 was identified in a screen for upregulated genes/sRNAs from EHEC attached to HeLa cells compared with EHEC grown in Dulbecco modified Eagle medium (DMEM).^105^ Esr055 is located in an intergenic region between *z3342* and *stx1B*, which encode a 9-O-acetyl-N-acetylneuraminic acid deacetylase, and the B subunit of Stx 1, respectively.^84^ Further characterization revealed that an *esr055* deletion increased the adherence of EHEC O157:H7 to HeLa cells. Additionally, greater numbers of the *esr055* deletion strain were recovered from the colons than from the ceca of infected mice.^84^ The expression of Esr055 is directly activated by the DeoR regulator, the expression of which is positively affected by DNA, which is significantly more abundant in the ileum than in the colon of mice.^84^ Finally, RNA-seq experiments comparing the expression of wild-type and *Δesr055* EHEC O157:H7 strains grown in vitro revealed the differential expression of over 400 genes, and led to the identification of 5 candidate genes (*z0568, z0974, z1356, z1926*, and *z5187*) as direct targets of Esr055 using informatics predictions.^84^

## Conclusions and future perspectives

Genomic comparison between EHEC O157:H7 and *E. coli* K-12 provided a broad array of whole genome-level information with biological and medical importance.^9,10^ The presence of a well-conserved 4.1-Mb sequence can be considered a chromosomal backbone in *E. coli* and numerous strain-specific DNA segments of foreign origins indicate how the two strains have been diversified from a common ancestral lineage. There is no doubt that the acquisition of OIs is critical for EHEC O157:H7 to evolve into successful human intestinal pathogens. Several comparative and epidemiological studies have indicated that EHEC O157:H7 descended from a nontoxigenic and less virulent EPEC O55:H7 ancestor via the following four sequential events: (1) acquisition of Stx2-containing bacteriophage (OI-45), (2) acquisition of the virulence plasmid pO157 and the genes encoding O antigens (OI-84), (3) acquisition of the Stx1-containing bacteriophage (OI-93), and (4) loss of the ability to ferment D-sorbitol and loss of beta-glucuronidase activity.^113,114^ Therefore, acquisition of these key OIs may promote the evolution of EHEC O157:H7 into the most virulent EHEC serotype, responsible for severer diseases and having low infection dose.^115^

Horizontally acquired OIs contribute to EHEC O157:H7 pathogenesis by providing various virulence elements, including virulence factors, effectors, as well as virulence regulatory proteins and sRNAs. The functions of several OIs were only established in recent years, revealing the association of more virulence elements with the virulence and pathogenesis of EHEC O157:H7. Nevertheless, the majority of OIs are comprised of genes of unknown function. For example, only the OI-9 encoded OvrB is required for bacterial adherence to host cells and the expression of LEE genes in EHEC O157:H7, as deletion of other OI-9 genes (*z0342, z0343, z0344, z0345, z0347*, and *z0348*) did not have a visible effect on EHEC O157:H7 virulence.^67^ Whether these OI-associated genes are redundant or involved in other bacterial processes needs to be further investigated. Moreover, the majority of current studies has mainly focused on investigating the relationship between OIs and the virulence of EHEC O157:H7. In contrast, studies on the functions of OIs associated with other physiological processes have been relatively limited. We previously reported that the OI-29 encoded *z0639* (also termed GmrA) was required for motility and flagellar synthesis in EHEC O157:H7.^116^ GmrA was shown to directly bind to the promoter of *fliA*, an RNA polymerase sigma factor, thereby regulating the FliA-controlled flagellar genes.^116^ Therefore, future studies should focus on the effect of OIs on other physiological functions, such as bacterial motility, acid resistance, and survival in adversity, which might facilitate an improved understanding of the EHEC pathogenicity and adaptation mechanisms to the host.

Diarrheic diseases caused by EHEC are an important public health problem worldwide. Outbreak surveillance data from the CDC have reported that EHEC O157:H7 alone results in more than 73,000 cases of illness, 2200 hospitalizations, and 60 deaths annually in the United States.^117^ Although several therapeutic strategies have been developed, including the use of antibiotics and vaccinations, there is no effective treatment for EHEC infections.^118^ Furthermore, the use of antibiotics has also been contraindicated, as antibiotic-induced bacterial cell lysis would lead to an increased release of Stxs.^119^ Therefore, highly effective measures for the prevention and control of EHEC O157:H7 infections are essential. Considering that the deletion of many OIs genes would significantly decrease EHEC O157:H7 adherence and expression of virulence genes both in vitro and in vivo, these virulence-related OIs genes might hence be used as potential targets for the development of novel therapeutics for the treatment of EHEC O157:H7 infections.

## Supplementary Material

Supplemental Material
